# Forest carbon in lowland Papua New Guinea: Local variation and the importance of small trees

**DOI:** 10.1111/aec.12187

**Published:** 2014-09-25

**Authors:** John B Vincent, Bridget Henning, Simon Saulei, Gibson Sosanika, George D Weiblen

**Affiliations:** 1 Plant Biological Sciences Graduate Program, University of MinnesotaSaint Paul, MN, 55108, USA; 2 Conservation Biology Graduate Program, University of MinnesotaSaint Paul, Minnesota, USA; 3 Bell Museum and Department of Plant Biology, University of MinnesotaSaint Paul, Minnesota, USA; 4 Papua New Guinea Forest Research InstituteLae, Papua New Guinea; 5 New Guinea Binatang Research CenterMadang, Papua New Guinea

**Keywords:** above-ground living biomass, forest carbon, Papua New Guinea, tropical rain forest

## Abstract

Efforts to incentivize the reduction of carbon emissions from deforestation and forest degradation require accurate carbon accounting. The extensive tropical forest of Papua New Guinea (PNG) is a target for such efforts and yet local carbon estimates are few. Previous estimates, based on models of neotropical vegetation applied to PNG forest plots, did not consider such factors as the unique species composition of New Guinea vegetation, local variation in forest biomass, or the contribution of small trees. We analysed all trees >1 cm in diameter at breast height (DBH) in Melanesia's largest forest plot (Wanang) to assess local spatial variation and the role of small trees in carbon storage. Above-ground living biomass (AGLB) of trees averaged 210.72 Mg  ha^−1^ at Wanang. Carbon storage at Wanang was somewhat lower than in other lowland tropical forests, whereas local variation among 1-ha subplots and the contribution of small trees to total AGLB were substantially higher. We speculate that these differences may be attributed to the dynamics of Wanang forest where erosion of a recently uplifted and unstable terrain appears to be a major source of natural disturbance. These findings emphasize the need for locally calibrated forest carbon estimates if accurate landscape level valuation and monetization of carbon is to be achieved. Such estimates aim to situate PNG forests in the global carbon context and provide baseline information needed to improve the accuracy of PNG carbon monitoring schemes.

## Introduction

Deforestation and forest degradation are one of the greatest sources of carbon emissions (approx. 17%) contributing to anthropogenic climate change (Stern 2007). The United Nations initiative to reduce emissions from deforestation and degradation (REDD+) aims to reward nations and stakeholders for forest carbon storage and sequestration (Angelsen 2008). REDD+ accounting requires accurate estimation of carbon storage under different land use options and associated changes in emission or sequestration due to changes in land use (Gibbs *et al*. 2007). Large variation among regions (e.g. sub-Saharan Africa estimated at 80 Mg C  ha^−1^, Latin America at 99 Mg C  ha^−1^, or Asia and Oceania at 137 Mg C  ha^−1^) necessitates local estimates of forest carbon (Saatchi *et al*. 2011).

Located in the Southwest Pacific Ocean directly north of Australia, the island of New Guinea is the world's largest tropical island and holds the third largest expanse of tropical forest in the world (Mittermeier *et al*. 1998). Composed of the eastern half of the island of New Guinea and surrounding islands, Papua New Guinea (PNG) is a heavily forested country with mainland forests totalling 33 million hectares of which 19 million hectares are lowland rainforest (Shearman *et al*. 2008; Shearman & Bryan 2011). This important carbon pool is remarkably intact relative to other equatorial forests but threatened by a deforestation rate of 1.4% per annum (Shearman *et al*. 2008). Forest carbon estimates for PNG have been contentious. Ground-based forest inventories have produced estimates of lowland rainforest carbon ranging from 111.34 Mg C  ha^−1^ (Bryan *et al*. 2010a) and 120.8 Mg C  ha^−1^ (Fox *et al*. 2010) to 169.9 Mg C  ha^−1^ (Bryan *et al*. 2010b) whereas estimates from remote sensing ranged 147–153 Mg C  ha^−1^ (Saatchi *et al*. 2011). Scaling such variation to the estimated 18.65 million hectares of lowland rainforest in PNG (Shearman & Bryan 2011) suggests that massive uncertainty is associated with the regional carbon stock. Differing estimates could have great economic consequences should monetary investment become available through REDD+ initiatives.Differing carbon estimates might either have a biological explanation or be attributed to methodological differences among studies or errors (Bryan *et al*. 2010a,b[Bibr b4],[Bibr b5]; Fox *et al*. 2010, 2011).

Forest dynamics, patterns of recruitment and mortality over time that result in turnover among individual trees, are also an important consideration. PNG forests are alleged to be more highly dynamic than other tropical forests (Johns 1986) as a result of extreme topography, unstable terrain, volcanic activity, frost, flood, fire and a substantial history of human use (Johns 1986; Filer *et al*. 2009; Fox *et al*. 2011). Such disturbances result in a heterogeneous landscape and a fine-grained matrix of spatial variation in forest cover where recently disturbed areas contain greater numbers of small, young trees than adjacent areas. We would expect more highly dynamic forests to store less carbon overall, with greater spatial variability and proportionally more carbon in small trees, compared to less dynamic forests. Indeed, studies have identified regional differences in above-ground biomass associated with tree stature and density of large trees (Feldpausch *et al*. 2012; Slik *et al*. 2013).

Where PNG forests fit in this global picture remains an open question. Considering the floristic affinity with Australia, carbon stocks might be expected to resemble the wet tropical forests of Northeast Queensland that appear to be exceptionally high compared to the global average (Bradford *et al*. 2014; Murphy *et al*. 2013). Carbon estimates in PNG have been based on forest inventory data using allometric equations and measurements of tree diameter at breast height (DBH), wood density and height from arrays of vegetation plots (Bryan *et al*. 2010b; Fox *et al*. 2010). Plots less than 1 ha in size are logistically practical but may fail to capture local spatial variation in carbon storage across the landscape due to topographical heterogeneity or forest dynamics (Bryan *et al*. 2010b; Fox *et al*. 2010). Forest inventory methods may also vary from plot to plot and simplifying assumptions can contribute further error to carbon estimates. For example, estimates often rely on species-specific wood densities gathered from global databases but intraspecific variation in wood density among regions makes measurements incorporating local estimates of wood density more accurate (Feldpausch *et al*. 2012). In addition, often only large trees (>10 cm DBH) are directly measured, leaving small trees (<10 cm DBH), woody vines, below ground biomass and non-living biomass to be estimated as a proportion of large tree biomass. Although there is evidence of regional variation in the proportion of carbon in different size-classes, local estimates often assume proportions based on studies from distant regions (Chave *et al*. 2005). In PNG, where it is possible that small trees are more frequent than in comparable forests elsewhere, the common assumption that small tree biomass (<10 cm DBH) is equivalent to 5% of large tree biomass (Lugo & Brown 1992; Chave *et al*. 2003), may not be applicable.

This study aims to characterize local variation in carbon storage in PNG lowland rainforests and to identify possible sources of variation among previous estimates. We used a single large (50 ha) forest plot with measurements of all trees over 1 cm DBH to examine local spatial variation and the contribution of small trees to above-ground forest carbon. The large size of the plot provides a carbon estimate that is robust to local spatial variation and which is useful for interpreting differences among previous estimates based on smaller plots. Extensive measurement of small trees (<10 cm) further enabled the discovery of a substantially greater contribution of small trees to overall forest carbon than was known from forests elsewhere. Incorporating this new information improves the overall accuracy of estimates for PNG.

## Methods

### Study site and data

The Wanang Forest Dynamics Plot (FDP) was established in 2009 in lowland rainforest in the Middle Ramu region of Madang Province, Papua New Guinea (PNG). The plot is gridded to 20 m by 20 m quadrats according to protocol developed by the Center for Tropical Forest Science (Condit 1995). Topography is characterized by a riparian area along the eastern edge of the plot, sloping steeply to a plateau on the western edge. Elevation ranges from approximately 90 to 190 m above sea level. Climate is aseasonal, averaging 25.8°C and 4000 mm precipitation with over 125 mm of precipitation in each month. Rainfall and temperature data were collected at the Swire PNG rainforest project field station located adjacent to the 50-ha plot. Rainfall data were collected from June 2011 to March 2014, with 25 complete months of data collected during this period. The temperature figure reported is a mean average of daily temperatures measured hourly from June 17, 2010 to April 24, 2012. Soils are a shifting mosaic of Entisols, Inceptisols and Alfisols, depending on time since soil disturbance (B.L. Turner, pers. comm. 2013). Vegetation is classified as lowland tropical wet mixed evergreen forest (Paijmans 1976) with no evidence of recent human disturbance.

During 2009 through 2013, every woody stem >1 cm DBH in the Wanang FDP was tagged, DBH measured, mapped and identified to species. The 50-ha plot included 288 204 stems. In total, we recorded 536 taxa including 33 unnamed morphospecies and 503 named species. Morphospecies refer to undescribed taxa for which there may be uncertainty in identification beyond the genus level. The dataset also included 15 067 trees that could not be assigned to a morphospecies or species.

### Wood density

Wood specific gravity was obtained either from a destructively sampled forest plot at Wanang for 208 species (Whitfeld 2011) or publically available databases in the case of 81 species (World Agroforestry Centre Wood density database; Eddowes 1977; Chave *et al*. 2005; Alonk 2009). In the case of taxa lacking published measurements, simple averages of wood gravity were calculated at the levels of genus and family from compiled wood density data. For example *Aglaia brownii* did not have a wood specific gravity value in our data set, so we assumed phylogenetic conservatism and used the average *Aglaia* wood specific gravity estimate. Likewise, if no genus level data were available, a family level average was used. If no family level data were available, a community-wide average for Wanang forest species was used. Wood specific gravity values were assigned as specifically as compiled information and taxonomic identifications allowed, assuming phylogenetic conservatism of wood specific gravity. We assigned wood specific gravity values to 289 species, 194 taxa were assigned a genus average value, 26 taxa were assigned a family average value, and the community average wood specific gravity was used for 27 taxa lacking taxonomic information.

### Above-ground biomass estimation

The allometric equation for tropical wet forests derived by Chave *et al*. (2005) was used to estimate above-ground living biomass: AGLB (in kg) = *ρ*^(−1.239 + 1.980 ln(^*^D^*^) + 0.207(ln(^*^D^*^))2 − 0.0281(ln(^*^D^*^))3)^, where *ρ* is wood specific gravity (g/cm^2^) and *D* is diameter (cm). Averages and 95% confidence intervals (CI) for AGLB and stem density were produced by 1000 bootstraps on 1-ha sub-plots to provide information on spatial variation in biomass (sensu Ngo *et al*. 2013). Species-level biomass estimates were obtained on a per hectare basis by dividing total biomass within a species by the total area sampled in hectares. We used a conventional conversion factor of 0.5 to convert from AGLB to carbon (Malhi *et al*. 2004; Fox *et al*. 2010). Root biomass was assumed to be 12% of AGLB following Bryan *et al*. (2010a). All calculations were performed in R v2.15. Bootstrap averages, confidence intervals were calculated in the package ‘boot’.

The relationship between AGLB and plot topography was explored using ordinary least squares regression. Topographic variables were calculated from plot survey data using the CTFS R package. Relationships between elevation, slope, convexity of quadrats and AGLB were explored in separate regression models. AGLB was log-transformed for normality and homoscedasticity. Significance was evaluated for each linear model at α = 0.05 and the explanatory value of models was assessed based on evaluation of the portion of variance explained (*r*^2^). A two-tailed *t*-test compared per-hectare small tree biomass (<10 cm) based on measurements of individuals >1 cm to estimates based on the common assumption that small trees constitute 5% of large tree biomass.

Extrapolations of AGLB to the entirety of the Wanang Conservation Area were also made. We obtained an estimate for the 10 770-ha area by the multiplying our per hectare biomass estimate by 10 770. We also multiplied the most extreme values among the 50-ha subplots by the total area to illustrate possible skew in extrapolated estimates should extremely high or low biomass per hectare be assumed. Extrapolated values were also multiplied by 0.5 to estimate above-ground carbon for Wanang.

## Results

### Biomass and carbon storage

**Figure 1:**
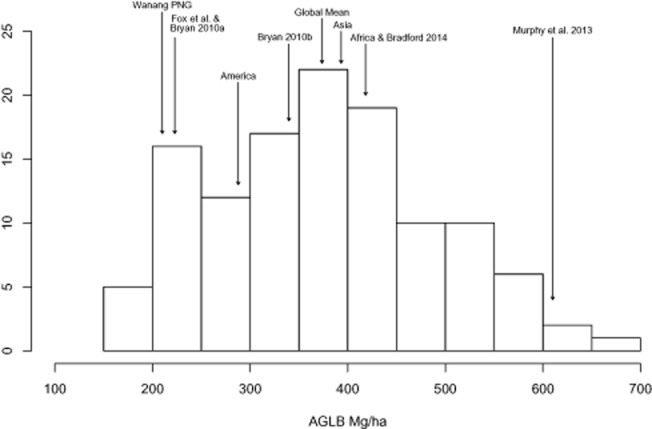
Histogram of tropical forest AGLB. Arrows indicate estimates of lowland rainforest AGLB from Bryan *et al*. (,b2010a), Fox *et al*. (2010), and for Wanang. A global mean (373.65 Mg  ha^−1^) and means for American (287.85 Mg  ha^−1^), African (418.28 Mg  ha^−1^) and Asian (393.25 Mg  ha^−1^) regional estimates derived from Slik *et al*. (2013). Australian estimates are provided from Bradford *et al*. (2014) and Murphy *et al*. (2013).

We estimated 210.7 (95% CI 191.1–226.9) Mg  ha^−1^ of above-ground living biomass (105.4 Mg C  ha^−1^) in trees >1 cm DBH (Table 1). Compared to other tropical lowland rainforests globally, our estimate suggests that PNG has relatively low biomass on a per hectare basis (Fig. 1). Our estimate of 210.7 Mg  ha^−1^ is much lower than a global mean estimate (373.7 Mg  ha^−1^) as well as estimates for lowland rainforests of the Americas (287.9 Mg  ha^−1^), Asia (393.24), Africa (393.3) (Slik *et al*. 2013) and Australia (513.6 Mg  ha^−1^) (Bradford *et al*. 2014; Murphy *et al*. 2013; Fig. 1).

**Table 1:** Comparison of PNG lowland primary forest biomass estimates

Biomass (Mg ha^−1^)	Our estimate	Fox *et al*. (2010)	Bryan *et al*. (2010b)
Large trees (>10 cm)	195.53	212.6	NA
Small trees (<10 cm)	15.19 (measured)	10.2 (calculated)	NA
AGLB	210.72	222.8	*339.74*
Roots (12% AGLB)	25.29	*26.74*	*46.33*
Total	236.01	*249.536*	*386.11*
Area studied	50 ha	10 ha	NA

Bryan *et al*. estimate was extracted from lowland forest sites included in their study. Bryan *et al.'s*, detailed calculation and plot methods were unavailable. Italics for inferred value calculated from description of methods in literature.

**Figure 2:**
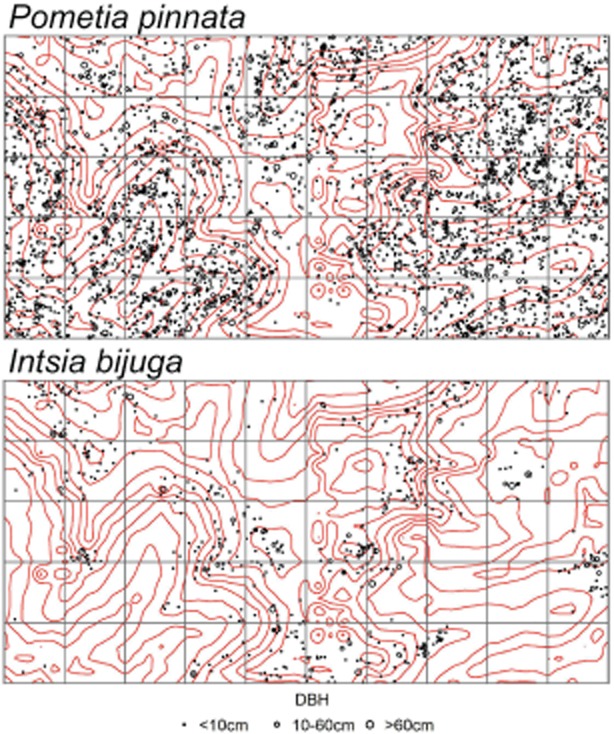
Tree distributions for the two most massive species in 30 ha of Wanang forest. The top panel shows *Intsia bijuga* (Fabaceae), closely associated with ridgetops. The bottom panel shows *Pometia pinnata* (Sapindaceae), dominant in ravine and riparian areas. Biomass in the plot is heavily influenced by *Intsia bijuga*, as can be seen comparing its stem distribution in Figure 2 and spatial variation in plot biomass in Figure 4.

We found small trees (1–10 cm DBH) averaging 5240 stems per hectare (95% CI 5100–5385) and accounting for 15.19 Mg  ha^−1^ AGLB (95% CI 14.8–15.6), or 7.2% of total AGLB in the plot as a whole (Table 2). The majority of AGLB in the Wanang plot was in the 10–70 cm DBH size-class (157.84 Mg  ha^−1^; 95% CI 148.4–167.1), making up 72.6% of total biomass (Table 2). There were rather few very large trees (>70 cm) in the Wanang plot, averaging only 5 per hectare (95% CI 4–6, Table 2). These few trees contributed disproportionately to AGLB, accounting for 37.69 Mg  ha^−1^ (95% CI 28.9–46.3) and 17.92% of total AGLB (Table 2). The top ten tree species in terms of biomass represented 36.3% of the total (Table 3). The most abundant tree in our plot, *Pometia pinnata*, accounted for 11.62% while *Intsia bijuga*, a valuable timber species, accounted for 4.74%. The top ten are among the few reaching sizes >70 cm at Wanang and trees this large are rather rare (Table 3, Fig. 2).

**Table 2:** Distribution of stems and biomass across size-classes

Size-class	Stems per hectare [95% CI]	AGLB (Mg ha^−1^) [95% CI]	% AGLB
1–10 cm	5240[5100–5385]	15.19[14.79–15.60]	7.21
10–70 cm	518[502–535]	157.84[148.38–167.08]	74.90
**≥**70 cm	5[4–6]	37.69[28.93–46.31]	17.89

**Table 3:** Ten species in 50 ha of Wanang forest with the highest above-ground living biomass (AGLB) and the percent of total biomass

Species (family)	AGLB (Mg ha^−1^)	% AGLB per hectare
*Pometia pinnata* (Sapindaceae)	24.45	11.62
*Intsia bijuga* (Fabaceae)	9.98	4.74
*Mastixiodendron pachyclados* (Rubiaceae)	7.72	4.67
*Celtis latifolia* (Cannabaceae)	7.17	3.41
*Pimelodendron amboinicum* (Euphorbiaceae)	6.49	3.09
*Gnetum gnemon* (Gnetaceae)	5.04	2.40
*Neonauclea obversifolia* (Rubiaceae)	3.93	1.87
*Vitex cofassus* (Lamiaceae)	3.52	1.67
*Erythrospermum candidum* (Salicaceae)	3.29	1.56
*Pterocarpus indicum* (Fabaceae)	3.28	1.26

### Local variation and small trees

**Figure 3:**
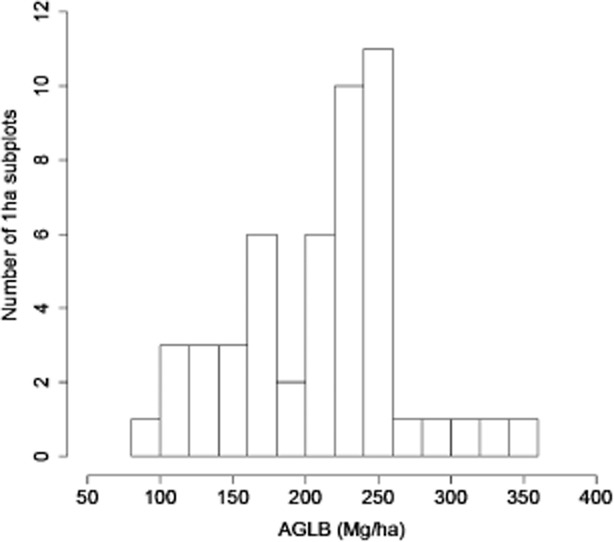
Histogram of 1-ha subset values in 50 ha of Wanang forest. The range of AGLB per hectare at Wanang ranged from 162.63 to 328.40 Mg  ha^−1^ with a mean of 228.74 and 95% CI of 214.38–241.58.

**Figure 4:**
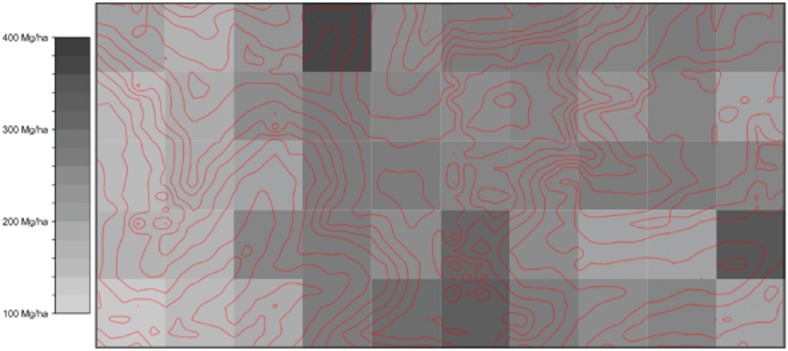
Spatial variation in AGLB in 50 ha of Wanang forest with red 10 m topographical contours. Grayscale colours indicate variation in AGLB in 1-ha subplots.

Variation in biomass among contiguous 50-ha subplots was considerable and ranged from 96.5 to 347.8 Mg  ha^−1^ (Fig. 3). Spatially auto-correlated patterns of variation in AGLB were not evident (Fig. 4), nor was AGLB correlated with elevation, slope, or convexity.

Estimating AGLB of small trees using the conventional assumption of 5% of trees < 10 cm DBH yielded an average of 9.8 Mg  ha^−1^ and was significantly less than our measured value (*t* = 12.18, d.f. = 49, *P* < 0.0001). Few studies have reported comparable biomass estimates including trees as small as 1 cm DBH (Table 4). Tropical Asian and American forests appear to exhibit considerable variation in the percentage of total biomass represented by small trees, ranging from 2.74% in Panama (Kirby & Potvin 2007) to 7.78% in Yasuni, Ecuador (Valencia *et al*. 2009).

**Table 4:** Comparison of biomass in trees <10 cm DBH

≤10 cm DBH AGLB (Mg ha^−1^)	% total AGLB	Stems per hectare	Location	Source
15.19	7.21%	5240	PNG	
11.58	4.22%	4092	Panama	Chave *et al*. (2003)
15.37	4.58%	5909	Singapore	Ngo *et al*. (2013)
20.6–21.2	7.52–7.78%	5132–5347	Ecuador	Valencia *et al*. (2009)
13.1	2.74%	NA	Panama	Kirby and Potvin (2007)

Results of our study are presented in the first row along with other studies that have measured biomass in small trees. Valencia *et al*. (2009) reported results from two censuses of the same plot.

### Carbon extrapolation and valuation

Our best estimate of above-ground biomass for the 10 770-ha Wanang Conservation Area was 2 465 000 Mg. This amount of biomass is equivalent to 1 135 000 Mg of carbon. Depending on assumptions, estimates ranged from 520 000 to 1 830 000 Mg C (Table 5).

**Table 5:** Comparison of five different carbon estimates extrapolated to the entirety of the Wanang Conservation Area (10 770 ha)

Carbon estimate	Wanang carbon (Mg)
Mean estimate	1 135 000
Low	520 000
High	1 873 000
Fox *et al*. (2010)	1 200 000
Bryan *et al*. (2010b)	1 830 000

## Discussion

Although Papua New Guinea is known to support significant expanses tropical forest, studies of PNG forest biomass and carbon storage are relatively few (Edwards & Grubb 1977; Bryan *et al*. 2010a,b[Bibr b4],[Bibr b5]; Fox *et al*. 2010, 2011). Previous estimates for lowland PNG rainforest AGLB are divergent (Bryan *et al*. 2010b; Fox *et al*. 2010), either agreeing closely with our estimate (Fox *et al*. 2010, 222.8 Mg  ha^−1^, Bryan *et al*. 2010a, 222.68 Mg  ha^−1^), or being much higher than ours, greater than the American estimate, and nearer to the global mean (Bryan *et al*. 2010b, 339.75). Assuming 12% root biomass, as did Bryan *et al*. (2010b), only elevated our estimate to 235.60 Mg  ha^−1^ (128.095 Mg C  ha^−1^, Table 1). We sought to improve on prior estimates by measuring local variation and the contribution of small trees to overall forest biomass. The inclusion of species-specific and site-specific wood density measures with a large, detailed and spatially explicit dataset allowed us to examine spatial heterogeneity and demographic patterns in PNG rainforest carbon as never before. Our results demonstrate that the estimates of Fox *et al*. (2010) and Bryan *et al*. (2010a) are generally robust to assumptions about wood density, small trees and spatial variation. We did not evaluate the accuracy of allometric equations in estimating forest biomass or include tree height as a parameter but our findings do improve the accuracy of PNG lowland forest biomass estimates and help to situate PNG forests in a global context.

### Comparing estimates

This study, in agreement with Fox *et al*. (2010) and Bryan *et al*. (2010a), found that PNG forest biomass islower than the global average for rainforests (Fig 1). The question is how much lower. The average for the Wanang plot (Table 2) closely matches that of Fox *et al*. (2010) and Bryan *et al*. (2010a) and is significantly lower than that of Bryan *et al*. (2010b), who included the Middle Ramu in their study. The Middle Ramu estimate from Bryan *et al*. (2010b) was obtained from a single destructively sampled hectare (Whitfeld *et al*. 2012b) located about 15 km from the Wanang FDP and in similar terrain. Considering that the Middle Ramu plot reported in Bryan *et al*. (2010b) was substantially more massive than the Wanang FDP average (320 Mg  ha^−1^ compared to 210.7 Mg  ha^−1^ total biomass), perhaps the divergent estimates of different authors might be explained by a bias toward locating the plot in more massive forests. Re-analysing data from the 1-ha destructively sampled plot (Whitfeld *et al*. 2012b) using the method of Bryan *et al*. (2010b) produced an estimate of 294.2 Mg  ha^−1^. This figure is much larger than our average per hectare AGLB estimate of 210.7, but does fall within the range of 96.5–347.8 Mg  ha^−1^ within the 50 ha FDP. This large value relative to our plot average illustrates the possible bias introduced by limited spatial sampling to characterize forest biomass.

The lesser biomass of PNG forests compared to the global average could be due to their dynamic nature (Swaine & Whitmore 1988). Although there have been no long-term studies of forest dynamics in PNG, recent observations suggest that PNG forests could have higher turnover than other forests around the world (J.B. Vincent, unpubl. data 2014). Forests with higher rates of tree turnover resulting from disturbance and successional processes will have greater spatial variability in structure and a higher proportion of biomass represented by small trees.

By including measurements of small trees, species-specific wood density measures and local spatial variation our estimate provides a unique perspective into above-ground carbon storage in PNG. Although our estimate applies only to lowland primary rainforest, this forest type covers a vast area of 18.65 million hectares in PNG (Shearman & Bryan 2011). Extrapolation to such scales calls for increased precision in biomass estimates. Our results show that consideration of spatial heterogeneity, forest dynamics and species-specific wood density measures can improve the accuracy of carbon estimates.

### Small trees

Forest succession theory predicts that a more dynamic forest will contain a higher proportion of biomass in small trees (Chazdon 2008). Shaped by demographic and successional processes, early secondary forests have been shown to store up to 20% of carbon in trees < 10 cm DBH compared to 5% in mature forests (Lugo & Brown 1992; Chave *et al*. 2003; Fox *et al*. 2010). It is an oversimplification to regard pristine forests as uniform in structure when in fact they represent a mosaic of successional patches according to the frequency, intensity and scale of natural disturbances over time. In lowland PNG, forest succession is associated with increasing biomass, species richness, phylogenetic diversity and functional complexity (Whitfeld *et al*. 2012a). Variation in topography, soils, climate and biotic interactions can influence disturbance regimes that are further propagated over time to spatial heterogeneity in stem size distributions and forest biomass. Undercutting of trees by rapid erosion, mud flows and slumps during periods of heavy rainfall may be a particularly important source of disturbance at Wanang where uplifted oceanic sediments form an unstable and highly dissected terrain of ridges, coves and ravines across much of the PNG lowlands (Loffler 1977). These observations are consistent with the idea that mature forests in PNG generally store a higher proportion of carbon in small trees than most lowland tropical rainforests.

Plot-based vegetation methods inevitably set a minimum diameter for stem tree measurements and, commonly, only trees > 10 cm DBH are measured. The popular assumption that biomass of trees < 10 cm DBH is equal to 5% of large tree biomass underestimated Wanang tree biomass by 4.4 Mg  ha^−1^. This error may seem small but extrapolation could propagate a minor discrepancy across millions of hectares and grossly underestimate forest carbon at larger scales. On the other hand, our assumptions about wood density may incorrectly estimated small tree biomass. Wood density is known to vary within trees (e.g. Wiemann & Williamson 1989; Swenson & Enquist 2008) and may also change with ontogeny. Destructive sampling of small trees will be required to evaluate potential bias in wood density assumptions with respect to size. The 5% assumption drawn from forests in other regions may be a reasonable simplifying assumption but not in lowland PNG. The only other quantification of small trees in PNG found 3% of above-ground biomass in small trees in a 0.24-ha mid-montane forest plot (Edwards & Grubb 1977), which is a rather small sample and certainly does not apply to lowland forests. Large trees are obviously the most influential as they store the majority of carbon (Slik *et al*. 2013), but it is important not to overlook regional variation in the contribution of small trees to overall biomass as it affects carbon accounting. Our study demonstrates that, despite small trees playing a relatively minor role in carbon storage, extrapolations are rather sensitive to assumptions about the distribution of biomass among tree size-classes.

### Local heterogeneity

Variation observed among subplots within 50 ha of contiguous forest (Fig. 3) supports the concept of mature forests as mosaics of forests in different phases of succession and biomass accumulation (Franklin *et al*. 2002; Coomes & Allen 2007). These findings demonstrate the importance of plot size in accurately assessing carbon storage (Chave *et al*. 2001, 2003). Nascimento and Laurance (2001) suggested that a relatively small number of satellite plots can accurately assess forest carbon and previous studies have focused on the importance of site selection to adequately represent regional variation (Bryan *et al*. 2010b). However, we would argue that local variation could be just as important as regional variation. Plot sites are often chosen based on the presence of large trees and undisturbed appearance (‘old-growth character’) that may result in selection of sites exhibiting high biomass relative to surrounding forest (Phillips *et al*. 2002). Measurements obtained from small plots, when extrapolated to larger scales, can dramatically affect estimates of carbon at the landscape level (Table 5). Large plots minimize bias and improve accuracy by integrating across successional stages, topography and other sources of local heterogeneity in biomass accumulation (Chave *et al*. 2003). Extreme heterogeneity of AGLB at Wanang (Fig. 2) suggests that the randomized placement of multiple small plots could be the most practical solution to avoiding bias. We would further argue that the improved accuracy gained from a large plot is marginal compared to the cost of a large census. Instead, we recommend effort to avoid bias in site selection and a sufficient number of small plots to approximate a landscape-level average.

### Limitations

Our study examined some of the methodological limitations of prior work including plot size, lack of small tree measurements and wood density assumptions. Further limitations that also apply to our own estimates include failure to incorporate height measurements or to validate the assumption that carbon represents 50% of AGLB (Martin & Thomas 2011). Inclusion of height measurements in allometric equations has been shown to significantly improve biomass estimates (Feldpausch *et al*. 2012). By definition, a single large forest plot does not capture regional variation and thus our estimates are not representative for PNG lowland forests as a whole but our findings suggest a number of steps that can be taken to improve regional estimates based on smaller plots. These include randomized site selection, increasing the contribution of small trees to overall biomass above 5%, and employing locally appropriate wood density information.

### Carbon extrapolation

We extrapolated our Wanang estimate and AGLB estimates from Fox *et al*. (2010) and Bryan *et al*. (2010b) for the sake of comparison. Carbon in the 10 770-ha Wanang Conservation Area forest was estimated at 1.2 million Mg. Had we endeavoured to obtain a value by approximation from the literature, our estimate would have varied by 50%, ranging from 1.2 to 1.8 million Mg (Table 5). Likewise, considering the spatial heterogeneity of Wanang forest biomass, we could have derived an estimate anywhere from 0.8 to 1.8 million Mg depending on which hectare we sampled.

As with any extrapolation, scaling estimates of forest biomass per hectare to a landscape level will propagate errors. The extrapolations required by schemes to monetize carbon and incentivize forest preservation for the purposes of reducing global atmospheric carbon concentrations should be regarded with great caution. Our results show substantial biomass variation in a contiguous forest that many would assume to be homogenous. Although large plots like Wanang are impractical for REDD+ implementation, we suggest that greater consideration be given to local spatial heterogeneity. Should projects such as REDD+ move past the pilot phase and into wider implementation it will be important to consider how errors associated with carbon estimates may impact economic reality.

## Conclusions

Carbon estimates are ideally generalizable across the landscape. The largest and most detailed measurement of a continuous forest in Melanesia suggests that PNG lowland rainforests contain less biomass per hectare than lowland tropical rainforests on average. This pattern could be due to the elevated disturbance and dynamism of lowland PNG forests, an explanation that has been suggested in the literature but remains untested. A recensus of the Wanang plot is needed to examine this possibility.

We also found a higher proportion of biomass in small trees than is typically assumed for lowland tropical rainforests. Our findings suggest that the carbon estimates reported by (Bryan *et al*. 2010a; Fox *et al*. 2010) are more accurate than those of (Bryan *et al*. 2010b). We conclude that randomized sampling, appropriate wood density information, and the contribution of small trees should be considered to ensure that estimates are as accurate as possible.
